# Genetic polymorphisms of enzyme proteins and transporters related to methotrexate response and pharmacokinetics in a Japanese population

**DOI:** 10.1186/s40780-016-0069-0

**Published:** 2016-12-09

**Authors:** Masayuki Hashiguchi, Mikiko Shimizu, Jun Hakamata, Tomomi Tsuru, Takanori Tanaka, Midori Suzaki, Kumika Miyawaki, Takeshi Chiyoda, Osamu Takeuchi, Jiro Hiratsuka, Shin Irie, Junya Maruyama, Mayumi Mochizuki

**Affiliations:** 1 Division for Evaluation and Analysis of Drug Information, Faculty of Pharmacy, Keio University, 1-5-30 Shibakoen, Minato-ku, Tokyo, 105-8512 Japan; 2 Department of Hygienic Chemistry, Faculty of Pharmacy, Keio University, 1-5-30 Shibakoen, Minato-ku, Tokyo, 105-8512 Japan; 3 PS Clinic, LTA Clinical Pharmacology Center, 6-18 Tenyacho, Hakata-ku, Fukuoka, 812-0025 Japan; 4 Sumida Hospital, LTA Clinical Pharmacology Center, 1-29-1 Honjo, Sumida-ku, Tokyo, 130-0004 Japan; 5 Division of Research, BioMedical Laboratory, Kitasato University Kitasato Institute Hospital, 5-9-1 Shirokane, Minato-ku, Tokyo, 108-8642 Japan; 6 Kyushu Clinical Pharmacology Research Clinic, LTA Clinical Pharmacology Center, 6-18 Tenyacho, Hakata-ku, Fukuoka, 812-0025 Japan

**Keywords:** Methotrexate, Genetic polymorphism, Healthy Japanese population, Rheumatoid arthritis, SNPs

## Abstract

**Background:**

Methotrexate (MTX) is currently the anchor drug widely used worldwide in the treatment of rheumatoid arthritis (RA). However, the therapeutic response to MTX has been shown to vary widely among individuals, genders and ethnic groups. The reason for this has been not clarified but it is considered to be partially due to several mechanisms in the cellular pathway of MTX including single-nucleotide polymorphisms (SNPs). The purpose of this study was to investigate the allelic frequencies in different ethnic and/or population groups in the 10 polymorphisms of enzyme proteins and transporters related to the MTX response and pharmacokinetics including MTHFR, TYMS, RFC1, FPGS, GGH, ABCB1, ABCC2 and ABCG2 in unrelated healthy Japanese adults and patients with RA.

**Methods:**

Ten polymorphisms, methylenetetrahydrofolate reductase (MTHFR) 1298, thymidylate synthase (TYMS) 3'-UTR, reduced folate carrier 1 (RFC1) 80 and−43, folypolyglutamyl synthase (FPGS) 1994, γ-glutamyl hydrolase (GGH) 452 and−401, the ABC transporters (ABCB1 3435, ABCC2 IVS23 + 56, ABCG2 914) of enzyme proteins and transporters related to MTX response and pharmacokinetics in 299 unrelated healthy Japanese adults and 159 Japanese patients with RA were investigated to clarify their contributions to individual variations in response and safety to MTX and establish personalized MTX therapy. SNPs were evaluated using real-time polymerase chain reaction (PCR).

**Results:**

Comparison of allelic frequencies in our study with other ethnic/population groups of healthy adults and RA patients showed significant differences in 10 polymorphisms among healthy adults and 7 among RA patients. Allelic frequencies of MTHFR 1298 C, FPGS 1994A and ABCB1 3435 T were lower in Japanese than in Caucasian populations and those of ABCC2 IVS23 + 56 C and ABCG2 914A were higher in Japanese than in Caucasian/European populations in both healthy adults and RA patients. Allelic frequencies of MTHFR 1298 C, GGH−401 T, ABCB1 3435 T, and ABCG2 914A were higher in healthy Japanese adults than in an African population, and those of RFC1 80A, RFC1−43C and ABCC2 IVS23 + 56 C in healthy Japanese adults were lower than in Africans. However, no significant differences were seen in the distribution of allelic frequencies between healthy Japanese adults and RA patients.

**Conclusion:**

The variations in allelic frequencies in different ethnic and/or population groups in healthy adults and RA patients may contribute to individual variations in MTX response and toxicity.

## Background

Rheumatoid arthritis (RA) is a systemic autoimmune inflammatory disease and its pathogenesis remains unclear. Although no curative therapy for RA has been established, the therapeutic goal is to delay symptom progression using disease-modifying anti-rheumatic drugs, gold preparations and biologics and achieve pain relief using nonsteroidal anti-inflammatory drugs (DMARDs). Among the therapeutic options, methotrexate (MTX) is currently the anchor drug widely used worldwide in the treatment of RA because it is inexpensive, effective and safe. In the 2014 Japanese therapeutic guidelines for RA treatment published by the Japan College of Rheumatology (JCR) [1], MTX was also cited as a first-line drug. However, the therapeutic response to MTX has been shown to vary widely among individuals, genders and ethnic groups [2, 3]. The reason for this has been not clarified but it is considered to be partially due to several mechanisms in the cellular pathway of MTX including single-nucleotide polymorphisms (SNPs) in transporters, glutamation, the folate pathway and adenosine pathway [2] such as reduced folate carrier 1 (RFC1), folypolyglutamyl synthase (FPGS), γ-glutamyl hydrolase (GGH), the ABC transporters ABCB1, ABCC2 and ABCG2, methylenetetrahydrofolate reductase (MTHFR), and thymidylate synthase (TYMS). We previously reported that the distribution of MTHFR C677T between black and Japanese populations, of TYMS 5'-UTR alleles between Caucasian or black and Japanese populations, and of TYMS 3'-UTR alleles between Caucasian and Japanese populations showed significant differences, as well as gender differences in TYMS 3'-UTR allelic frequency in Japanese [3].

Up to the present, a clear genetic difference has not been confirmed between healthy individuals and RA patients which would indicate susceptibility to RA, as such genetic variations do for many other diseases. There are few data on the possible genetic differences in RA, and therefore, it is important to clarify them to obtain useful information from the viewpoint of human genetics and/or population genetics, leading to the establishment of personalized medicine for those susceptible to the development of RA.

The purpose of this study was to investigate the allelic frequencies in different ethnic and/or population groups in the 10 polymorphisms of enzyme proteins and transporters related to MTX response and pharmacokinetics including MTHFR, TYMS, RFC1, FPGS, GGH, ABCB1, ABCC2 and ABCG2 in unrelated healthy Japanese adults and patients with RA.

## Methods

### Volunteers

A total of 299 unrelated healthy Japanese adult volunteers (148 men and 151 women) were recruited from Sumida Hospital (Tokyo) and 159 unrelated Japanese adult patients with RA (40 men and 119 women) admitted to PS Clinic (Fukuoka, Kyushu) were enrolled in this study. All were older than 20 years of age. MTX was administered to 99 (62.3%) of 159 RA patients, while 43 (27%) were receiving DMARDs, and 55 (34.6%) biologics as combination drugs. The daily dosage of MTX was 4 mg (3 patients), 6 mg (11 patients), 8 mg (75 patients), 10 mg (7 patients), and 12 mg (3 patients).

### DNA extraction

For genetic analysis, a 5-mL peripheral blood sample was obtained from each study participant using the standard venipuncture technique. The samples were placed in tubes containing ethylenediaminetetraacetic disodium salt (EDTA 2Na) and stored at−20 °C until DNA extraction. DNA was extracted using the Wizard Genomic DNA Purification Kit (Promega Corporation, WI, USA) following the manufacturer’s instructions. Total genomic DNA was quantified and its purity and integrity were analyzed using the NanoDrop 1000 spectrophotometer v3.7 (Thermo Scientific, Wilmington, DE, USA).

### Allele genotyping

Genotyping of alleles of MTHFR 1298A > C (rs1801131), TYMS 3'-UTR (−6/+6) (rs16430), RFC1 80G > A (rs1051266), RFC1−43 T > C (rs1131596), FPGS 1994G > A (rs10106), GGH 452C > T (rs11545078), GGH 401C > T (rs3758149), ABCB1 3435C > T (rs1045642), ABCC2 IVS23 + 56 T > C (rs4148396), and ABCG2 914C > A (rs2231142) was performed using the TaqMan SNP Genotyping Assay from Applied Biosystems (Foster City, CA, USA) with fluorogenic binding probes. PCR amplification with the real-time PCR method was performed in 25 μL of reaction mixture including genomic DNA 20 ng (60 ng for FPGS 1994G > A), 0.63 μL of 40 × TaqMan SNP Genotyping Assay Mix (0.32 μL for RFC1 80G > A), and TaqMan Universal PCR Master Mix 12.5 μL. The PCR reaction conditions were: initial denaturation for 10 min at 95 °C; 40 cycles at 92 °C/15 s; and annealing and extension for 1 min at 60 °C (55 °C for FPGS 1994G > A). For genotyping of alleles of ABCB1 3435C > T and ABCG2 914C > A, the PCR reaction conditions were: initial denaturation for 10 min at 95 °C; 50 cycles at 92 °C/15 s; and annealing and extension for 1 min at 60 °C. The PCR system used was the StepOnePlus real-time PCR system (Applied Biosystems).

### Assessment of ethnic or population and gender differences

We identified eligible comparative studies by searching MEDLINE (1966–May 2016), Embase (1974–May 2016), the Cochrane Library (Issue 1 of 12, January 2016), Japana Centra Revuo Medicina (Ichushi) (1981–May 2016), and the database of the HapMap Project (accessed in May 2016) [4] on the assumption that it contained healthy adult data. The search terms were MTHFR, methylenetetrahydrofolate reductase, TS, TYMS, thymidylate synthase, dihydrofolate reductase, RFC1, reduced folate carrier 1, Solute carrier family 19 (folate transporter) member 1, SLC19A1, folylpolyglutamate synthase, FPGS, gamma-glutamyl hydrolase, GGH, ATP-binding Cassette Sub-family B Member 1, ABCB1, ATP-binding cassette sub-family C member 2, ABCC2, ATP-binding cassette transporter G2 and ABCG2, in combination with genotype and polymorphism. All languages were included. Additionally, a manual search of reference listings from all of the articles retrieved from the electronic databases was performed. Selection criteria were case–control, cross-sectional or prospective cohort studies, which assessed any allelic frequencies of MTHFR 1298 A > C, TYMS 3'-UTR 6-bp deletion, RFC1 80 G > A, RFC−43 C > T, FPGS 1994 G > A, GGH 452 C > T, GGH−401C > T, ABCB 3435 C > T, ABCC2 IVS23 + 56 T > C, and ABCG2 914 C > A in healthy adults and RA patients and in which ethnic group was clearly identified. In case–control studies, only the allelic frequencies of the healthy adult control group were extracted. If we could not identify the ethnic group in the literature, population groups were assumed to be from the country of residence of the lead author.

### Statistical analysis

Comparisons of ethnic and gender differences in the distribution of allelic frequencies among genotypes, and/or gender differences and tests for Hardy-Weinberg equilibrium were carried out using the chi-square test. A *p*-value of <0.05 was considered to represent a significant difference in all statistical analyses. All statistical analyses were performed using JMP 12.0.1 software (SAS Institute Inc., Cary, NC, USA).

## Results

### Allelic frequencies in healthy adult populations and RA patients

Among the 298 studies retrieved from the electronic databases and those retrieved from their references by manual search, a total of 14,000 healthy adults from 16 studies [4–19] and 4284 RA patients from 25 studies [5, 6, 9, 14, 20–40], involving HapMap Projects [4] that described Japanese, Asian, European and African groups, were included in the comparative analysis (Tables 1 and 2). The number of individuals and published genotype frequency data for each ethnic/population group and comparisons with our Japanese allelic frequency results are shown in Tables 1 and 2.

**Table 1:** Comparison of distribution of MTX-related enzyme gene and transporter polymorphisms among ethnic or population group in healthy adults

Ethnic/population group	*n*	Genotype frequency (*n*)			HWE	Allele frequency (%)		*p*-value	Reference No.
MTHFR 1298		A/A	A/C	C/C		A	C		
Japanese (Our study)	299	206 (68.9)	84 (28.1)	9 (3.0)	0.902	83	17		
Japanese	477	316 (66.3)	146 (30.6)	15 (3.1)	0.707	82	18	0.7430	[4–6]
Asian	176	114 (64.8)	54 (30.7)	8 (4.5)	0.623	80	20	0.5310	[4]
Caucasian	1315	636 (48.3)	548 (41.7)	131 (10.0)	0.418	69	31	< 0.0001	[4, 7, 8]
African	346	270 (78.0)	74 (21.4)	2 (0.6)	0.198	89	11	0.0060	[4]
TYMS 3'-UTR		−6/−6	−6/+6	+6/+6		−6	+6		
Japanese (Our study)	299	127 (42.5)	137 (45.8)	35 (11.7)	0.833	65	35		
Japanese	239	106 (44.4)	114 (47.7)	19 (7.9)	0.123	68	32	0.3540	[6, 9]
RFC1 80		G/G	G/A	A/A		G	A		
Japanese (Our study)	299	52 (17.4)	155 (51.8)	92 (30.8)	0.336	43	57		
Japanese	472	90 (19.1)	228 (48.3)	154 (32.6)	0.731	43	57	0.6260	[4, 10]
Asian	168	46 (27.4)	76 (45.2)	46 (27.4)	0.217	50	50	0.0390	[4]
Caucasian	1203	363 (30.2)	585 (48.6)	255 (21.2)	0.498	55	45	< 0.0001	[4, 7, 8, 11]
African	249	33 (13.3)	92 (36.9)	124 (49.8)	0.020	32	68	< 0.0001	[4, 11]
RFC1−43		T/T	T/C	C/C		T	C		
Japanese (Our study)	299	52 (17.4)	155 (51.8)	92 (30.8)	0.336	43	57		
Japanese	172	34 (19.8)	84 (48.8)	54 (31.4)	0.897	44	56	0.7623	[4]
Asian	164	44 (26.8)	78 (47.6)	42 (25.6)	0.533	51	49	0.0520	[4]
Caucasian	226	64 (28.3)	128 (56.6)	34 (15.1)	0.021	57	43	< 0.0001	[4]
African	220	20 (9.1)	82 (37.3)	118 (53.7)	0.299	28	72	< 0.0001	[4]
FPGS 1994		G/G	G/A	A/A		G	A		
Japanese (Our study)	299	140 (46.8)	128 (42.8)	31 (10.4)	0.828	68	32		
Japanese	170	74 (43.5)	74 (43.5)	22 (13.0)	0.606	65	35	0.6390	[4]
Asian	630	315 (50.0)	251 (39.8)	64 (10.2)	0.185	70	30	0.6490	[4, 12]
Caucasian	226	30 (13.3)	104 (46.0)	92 (40.7)	0.943	36	64	< 0.0001	[4]
African	226	34 (15.0)	118 (52.2)	74 (32.8)	0.241	41	59	< 0.0001	[4]
GGH 452		C/C	C/T	T/T		C	T		
Japanese (Our study)	299	249 (83.3)	47 (15.7)	3 (1.0)	0.641	91	9		
Japanese	525	426 (81.1)	96 (18.3)	3 (0.6)	0.331	90	10	0.5180	[4, 13]
Asian	564	453 (80.3)	104 (18.5)	7 (1.2)	0.710	90	10	0.5680	[4, 12]
Caucasian	209	155 (74.2)	49 (23.4)	5 (2.4)	0.633	86	14	0.0350	[4, 7]
African	332	284 (85.5)	44 (13.3)	4 (1.2)	0.135	92	8	0.6650	[4]
GGH−401		C/C	C/T	T/T		C	T		
Japanese (Our study)	299	175 (58.5)	110 (36.8)	14 (4.7)	0.531	77	23		
Japanese	86	34 (39.5)	42 (48.8)	10 (11.7)	0.583	64	36	0.0031	[4]
Asian	90	54 (60.0)	32 (35.6)	4 (4.4)	0.786	78	22	0.9690	[4]
Caucasian	120	54 (45.0)	58 (48.3)	8 (6.7)	0.145	69	31	0.0421	[4]
African	120	90 (75.0)	26 (21.7)	4 (3.3)	0.232	86	14	0.0053	[4]
ABCB1 3435		C/C	C/T	T/T		C	T		
Japanese (Our study)	299	94 (31.4)	156 (52.2)	49 (16.4)	0.242	58	42		
Japanese	716	239 (33.4)	357 (49.9)	120 (16.7)	0.494	58	42	0.7850	[4, 9, 14–18]
Asian	84	32 (38.1)	34 (40.5)	18 (21.4)	0.125	58	42	0.1606	[4]
Caucasian	226	34 (15.0)	126 (55.8)	66 (29.2)	0.038	43	57	< 0.0001	[4]
African	226	178 (78.8)	46 (20.4)	2 (0.8)	0.605	89	11	< 0.0001	[4]
ABCC2 IVS23 + 56		T/T	T/C	C/C		T	C		
Japanese (Our study)	299	25 (8.4)	103 (34.4)	171 (57.2)	0.099	26	74		
Japanese	172	6 (3.5)	64 (37.2)	102 (59.3)	0.289	22	78	0.0971	[4]
Asian	86	4 (4.7)	44 (51.1)	38 (44.2)	0.048	30	70	0.0174	[4]
Caucasian	226	28 (12.4)	104 (46.0)	94 (41.6)	0.926	35	65	0.0018	[4]
African	226	4 (1.8)	88 (38.9)	134 (59.3)	0.014	21	79	0.0020	[4]
ABCG2 914		C/C	C/A	A/A		C	A		
Japanese (Our study)	299	166 (55.5)	111 (37.1)	22 (7.4)	0.565	74	26		
Japanese	4240	1999 (47.1)	1827 (43.1)	414 (9.8)	0.908	69	31	0.0170	[4, 14, 19]
Asian	176	90 (51.1)	70 (39.8)	16 (9.1)	0.655	71	29	0.6020	[4]
Caucasian	346	272 (78.6)	70 (20.2)	4 (1.2)	0.831	89	11	< 0.0001	[4]
African	344	342 (99.4)	2 (0.6)	0 (0)	0.957	100	0	< 0.0001	[4]

Values are given as *n* (%). *HWE*: *p*-value for chi-square test for agreement with Hardy-Weinberg equilibrium. *p*-value; comparison our study (Japanese) with each ethnic/population group

*MTHFR*: methylenetetrahydrofolate reductase, *TYMS*: thymidylate synthase, *RFC1*: reduced folate carrier 1, *FPGS*: folypolyglutamayl synthase, *GGH*: γ-glutamyl hydrolase, *ABCB1*: ATP binding cassette subfamily B member 1, *ABCC2*: ATP binding cassette subfamily C member 2, *ABCG2*: ATP binding cassette subfamily G member 2

**Table 2:** Comparison of distribution of MTX-related enzyme gene and transporter polymorphisms among ethnic or population group in the patients with rheumatoid arthritis

Ethnic/Population group	*n*	Genotype frequency (*n*)			HWE	Allele frequency (%)		*p*-value	Reference No.
MTHFR 1298		A/A	A/C	C/C		A	C		
Japanese (Our study)	159	109 (68.6)	42 (26.4)	8 (5.0)	0.149	82	18		
Japanese	357	232 (65.0)	107 (30.0)	18 (5.0)	0.224	80	20	0.7070	[5, 6, 20, 21]
Chinese	93	63 (67.7)	29 (31.2)	1 (1.1)	0.237	83	27	0.1690	[22]
Caucasian	1828	809 (44.3)	785 (42.9)	234 (12.8)	0.045	66	34	*p*<0.0001	[23–31]
African-American	138	102 (74.0)	35 (25.0)	1 (1.0)	0.278	87	13	0.0614	[26]
TYMS 3'-UTR		−6/−6	−6/+6	+6/+6		−6	+6		
Japanese (Our study)	159	72 (45.3)	67 (42.1)	20 (12.6)	0.477	66	34		
Japanese	409	173 (42.3)	193 (47.2)	43 (10.5)	0.314	66	34	0.5180	[6, 9, 32]
Caucasian	98	10 (10.2)	37 (37.8)	51 (52.0)	0.053	29	71	< 0.0001	[24]
RFC1 80		G/G	G/A	A/A		G	A		
Japanese (Our study)	159	34 (21.4)	74 (46.5)	51 (32.1)	0.461	45	55		
Japanese	681	135 (19.8)	348 (51.1)	198 (29.1)	0.421	45	55	0.5820	[5, 9, 20, 21, 32, 33]
Caucasian	611	187 (30.6)	275 (45.0)	149 (24.4)	0.017	53	47	0.0360	[24, 34–36]
RFC1−43		T/T	T/C	C/C		T	C		
Japanese (Our study)	159	34 (21.4)	74 (46.5)	51 (32.1)	0.461	45	55		
Caucasian	106	39 (36.8)	47 (44.3)	20 (18.9)	0.388	59	41	0.0760	[34]
FPGS 1994		G/G	G/A	A/A		G	A		
Japanese (Our study)	159	70 (44.0)	73 (45.9)	16 (10.1)	0.632	67	33		
Caucasian	205	42 (21.0)	99 (48.0)	64 (31.0)	0.743	45	55	< 0.0001	[37]
GGH 452		C/C	C/T	T/T		C	T		
Japanese (Our study)	159	134 (84.3)	22 (13.8)	3 (1.9)	0.081	91	9		
Japanese	142	129 (90.8)	13 (9.2)	0 (0.0)	0.568	95	5	0.1070	[20, 21]
Caucasian	571	479 (83.9)	91 (15.9)	1 (0.2)	0.119	92	8	0.0300	[27, 37, 38]
GGH−401		C/C	C/T	T/T		C	T		
Japanese (Our study)	159	88 (55.3)	62 (39.0)	9 (5.7)	0.654	75	25		
Japanese	257	169 (65.8)	78 (30.3)	10 (3.9)	0.790	81	19	0.1010	[21, 32]
ABCB1 3435		C/C	C/T	T/T		C	T		
Japanese (Our study)	159	54 (34.0)	82 (51.6)	23 (14.4)	0.363	60	40		
Japanese	174	61 (35.0)	80 (46.0)	33 (19.0)	0.460	58	42	0.4570	[9, 20]
Caucasian	769	178 (23.1)	381 (49.5)	210 (27.3)	0.838	48	52	0.0010	[27, 28, 36, 39]
ABCC2 IVS23 + 56		T/T	T/C	C/C		T	C		
Japanese (Our study)	159	6 (3.8)	55 (34.6)	98 (61.6)	0.614	21	79		
Caucasian	309	122 (39.5)	149 (48.2)	38 (12.3)	0.467	64	36	< 0.0001	[27, 40]
ABCG2 914		C/C	C/A	A/A		C	A		
Japanese (Our study)	159	77 (48.4)	67 (42.1)	15 (9.5)	0.939	69	31		
Japanese	55	30 (54.5)	20 (36.4)	5 (9.1)	0.537	73	27	0.7230	[14]
Caucasian	190	149 (78.4)	40 (21.1)	1 (0.5)	0.330	89	11	< 0.0001	[27]

Values are given as *n* (%). *HWE*: *p*-value for chi-square test for agreement with Hardy-Weinberg equilibrium. *p*-value; comparison our study (Japanese) with each ethnic/population group

*MTHFR*: methylenetetrahydrofolate reductase, *TYMS*: thymidylate synthase, *RFC1*: reduced folate carrier 1, *FPGS*: folypolyglutamayl synthase, *GGH*: γ-glutamyl hydrolase, *ABCB1*: ATP binding cassette subfamily B member 1, *ABCC2*: ATP binding cassette subfamily C member 2, *ABCG2*: ATP binding cassette subfamily G member 2

The distributions of the RFC1 80G > A allele in African [4, 11] in healthy adults and Caucasian [24, 34–36] in RA patients, RFC1−43 T > C allele in Caucasian [4] in healthy adults, ABCB1 3435 C > T allele in Caucasian [4] in healthy adults, and ABCC2 IVS23 + 56 T > C allele in Asian [4] and African [4] healthy adults were not in agreement with Hardy-Weinberg equilibrium. The distributions of the MTHFR 1298 A > C allele in Caucasian [23–31] in RA patients were also not in agreement with Hardy-Weinberg equilibrium.

Statistically significant differences were found in the distributions of the MTHFR 1298A > C, RFC1 80 G > A, RFC−43 T > C, FPGS 1994 G > A, GGH−401 C > T, ABCB1 3435 C > T, ABCC2 IVS23 + 56 T > C, and ABCG 914 C > A alleles in healthy adults between our Japanese population and Caucasians and Africans. Statistically significant differences were found in the distributions of the RFC1 80 G > A and ABCC2 IVS23 + 56 T > C alleles in healthy adults between our Japanese population and Asians (Table 1).

Statistically significant differences were found in the distributions of the MTHFR 1298A > C, TYMS 3'-UTR−6 > +6, RFC1 80 G > A, FPGS 1994 G > A, GGH 452 C > T, ABCB1 3435 C > T, ABCC2 IVS23 + 56 T > C, and ABCG 914 C > A alleles in RA patients between our Japanese population and Caucasians (Table 2).

### Allelic frequencies and gender differences in frequencies in healthy Japanese adults

The distribution of MTHFR 1298A > C, and TYMS 3'-UTR (−6/+6), RFC1 80G > A, RFC1−43 T > C, FPGS 1994G > A, GGH 452C > T, GGH−401C > T, ABCB1 3435C > T, ABCC2 IVS 23 + 56 T > C, and ABCG2 914C > A polymorphisms in 299 healthy Japanese adults are summarized in Table 3. The proportions of genotypes at each site for their transporter carrier proteins and enzymes were generally in agreement with Hardy-Weinberg equilibrium. Therefore, the proportions at each site followed Mendelian principles. Only three genotype combination patterns were found for RFC1 80G > A and RFC1−43 T > C, consisting of 80G/G,−43 T/T genotype, 80G/A,−43 T/C genotype, and 80A/A,−43C/C genotype, respectively and a strong linkage disequilibrium was observed in healthy Japanese adults.

**Table 3:** Distribution of MTX-related enzyme gene and transporter polymorphisms in healthy Japanese adults and patients with rheumatoid arthritis

	Healthy Japanese adults							Patients with rheumatoid arthritis							*p*-value^**^
	Genotype			Allele frequency (%)		*p*-value^*^	HWE	Genotype			Allele frequency (%)		*p*-value^*^	HWE	
MTHFR 1298	A/A	A/C	C/C	A	C			A/A	A/C	C/C	A	C			
Total	206 (68.9)	84 (28.1)	9 (3.0)	83	17		0.902	109 (68.6)	42 (26.4)	8 (5.0)	82	18		0.149	
Men	107 (72.3)	39 (26.3)	2 (1.4)	85	15		0.457	33 (82.5)	6 (15.0)	1 (2.5)	90	10		0.292	
Women	99 (65.6)	45 (29.8)	7 (4.6)	80	20	0.1611	0.522	76 (63.9)	36 (30.2)	7 (5.9)	79	21	0.0736	0.334	0.5478
TYMS 3'-UTR	−6/−6	−6/+6	+6/+6	−6	+6			−6/−6	−6/+6	+6/+6	-6	+6			
Total	127 (42.5)	137 (45.8)	35 (11.7)	65	35		0.833	72 (45.3)	67 (42.1)	20 (12.6)	66	34		0.478	
Men	64 (43.2)	66 (44.6)	18 (12.2)	66	34		0.877	20 (50.0)	10 (25.0)	10 (25.0)	62	38		0.003	
Women	63 (41.7)	71 (47.0)	17 (11.3)	65	35	0.9099	0.653	52 (43.7)	57 (47.9)	10 (8.4)	68	32	0.0064	0.304	0.7516
RFC1 80	G/G	G/A	A/A	G	A			G/G	G/A	A/A	G	A			
Total	52 (17.4)	155 (51.8)	92 (30.8)	43	57		0.336	34 (21.4)	74 (46.5)	51 (32.1)	45	55		0.461	
Men	17 (11.5)	80 (54.0)	51 (34.5)	39	61		0.086	9 (22.5)	18 (45.0)	13 (32.5)	45	55		0.565	
Women	35 (23.2)	75 (49.7)	41 (27.1)	48	52	0.0226	0.951	25 (21.0)	56 (47.1)	38 (31.9)	45	55	0.9698	0.605	0.47
RFC1−43	T/T	T/C	C/C	T	C			T/T	T/C	C/C	T	C			
Total	52 (17.4)	155 (51.8)	92 (30.8)	43	57		0.336	34 (21.4)	74 (46.5)	51 (32.1)	45	55		0.461	
Men	17 (11.5)	80 (54.0)	51 (34.5)	39	61		0.086	9 (22.5)	18 (45.0)	13 (32.5)	45	55		0.565	
Women	35 (23.2)	75 (49.7)	41 (27.1)	48	52	0.0226	0.951	25 (21.0)	56 (47.1)	38 (31.9)	45	55	0.9698	0.605	0.47
FPGS 1994	G/G	G/A	A/A	G	A			G/G	G/A	A/A	G	A			
Total	140 (46.8)	128 (42.8)	31 (10.4)	68	32		0.828	70 (44.0)	73 (45.9)	16 (10.1)	67	33		0.632	
Men	76 (51.3)	62 (41.9)	10 (6.8)	72	28		0.577	13 (32.5)	22 (55.0)	5 (12.5)	60	40		0.356	
Women	64 (42.4)	66 (43.7)	21 (13.9)	64	36	0.0775	0.550	57 (47.9)	51 (42.9)	11 (9.2)	69	31	0.2291	0.933	0.8131
GGH 452	C/C	C/T	T/T	C	T			C/C	C/T	T/T	C	T			
Total	249 (83.3)	47 (15.7)	3 (1.0)	91	9		0.641	134 (84.3)	22 (13.8)	3 (1.9)	91	9		0.081	
Men	124 (83.8)	21 (14.2)	3 (2.0)	91	9		0.079	34 (85.0)	5 (12.5)	1 (2.5)	91	9		0.169	
Women	125 (82.8)	26 (17.2)	0 (0.0)	91	9	0.097	0.247	100 (84.0)	17 (14.3)	2 (1.7)	91	9	0.9167	0.221	0.6543
GGH−401	C/C	C/T	T/T	C	T			C/C	C/T	T/T	C	T			
Total	175 (58.5)	110 (36.8)	14 (4.7)	77	23		0.531	88 (55.3)	62 (39.0)	9 (5.7)	75	25		0.654	
Men	91 (61.5)	49 (33.1)	8 (5.4)	78	22		0.679	19 (47.5)	19 (47.5)	2 (5.0)	71	29		0.313	
Women	84 (55.6)	61 (40.4)	6 (4.0)	76	24	0.3969	0.210	69 (58.0)	43 (36.1)	7 (5.9)	76	24	0.4479	0.930	0.7742
ABCB1 3435	C/C	C/T	T/T	C	T			C/C	C/T	T/T	C	T			
Total	94 (31.4)	156 (52.2)	49 (16.4)	58	42		0.242	54 (34.0)	82 (51.6)	23 (14.4)	60	40		0.363	
Men	45 (30.4)	80 (54.1)	23 (15.5)	57	43		0.199	16 (40.0)	19 (47.5)	5 (12.5)	64	36		0.861	
Women	49 (32.5)	76 (50.3)	26 (17.2)	58	42	0.7955	0.708	38 (31.9)	63 (52.9)	18 (15.1)	58	42	0.6454	0.328	0.7955
ABCC2 IVS23 + 56	T/T	T/C	C/C	T	C			T/T	T/C	C/C	T	C			
Total	25 (8.4)	103 (34.4)	171 (57.2)	26	74		0.099	6 (3.8)	55 (34.6)	98 (61.6)	21	79		0.614	
Men	4 (2.7)	51 (34.5)	93 (62.8)	20	80		0.333	1 (2.5)	17 (42.5)	22 (55.0)	24	76		0.273	
Women	21 (13.9)	52 (34.4)	78 (51.7)	31	69	0.0009	0.016	5 (4.2)	38 (31.9)	76 (63.9)	20	80	0.4574	0.928	0.1422
ABCG2 914	C/C	C/A	A/A	C	A			C/C	C/A	A/A	C	A			
Total	166 (55.5)	111 (37.1)	22 (7.4)	74	26		0.565	77 (48.4)	67 (42.2)	15 (9.4)	69	31		0.939	
Men	78 (52.7)	57 (38.5)	13 (8.8)	72	28		0.579	19 (47.5)	16 (40.0)	5 (12.5)	68	32		0.576	
Women	88 (58.3)	54 (35.7)	9 (6.0)	76	24	0.5003	0.852	58 (48.7)	51 (42.9)	10 (8.4)	70	30	0.7525	0.796	0.3325

Values are given as *n* (%)

*HWE*: *p*-value for chi-square test for agreement with Hardy-Weinberg equilibrium. *MTHFR*: methylenetetrahydrofolate reductase, *DHFR*: dihydrofolate reductase, *TYMS*: thymidylate synthase, *RFC1*: reduced folate carrier 1, *FPGS*: folypolyglutamayl synthase, *GGH*: γ-glutamyl hydrolase, *ABCB1*: ATP binding cassette subfamily B member 1, *ABCC2*: ATP binding cassette subfamily C member 2, *ABCG2*: ATP binding cassette subfamily G member 2

^*^ *p*-value: comparison of men and women

^**^ *p*-value: comparison of healthy adults and RA patients

Gender differences in the allelic frequencies are also summarized in Table 3. Allelic frequencies of RFC1 80G > A and RFC1−43 T > C were 61% in men and 52% in women for the A and C alleles and those of ABCC2 IVS23 + 56 T > C were 80% in men and 69% in women for the C allele. These differences in distribution were statistically significant (*p* = 0.0226 for RFC1 80G > A and RFC1−43 T > C, *p* = 0.0009 for ABCC2 IVS23 + 56 T > C, chi-square test). No gender differences in the distribution of other genotypes were observed (Table 3), although a tendency for a difference by gender was noted in the distribution of FPGS 1994 G > A (*p* = 0.0775, chi-square test) and GGH 452 C > T (*p* = 0.097, chi-square test).

### Allelic frequencies and gender differences in frequencies in Japanese RA patients

The distributions of MTHFR 1298A > C, and TYMS 3'-UTR (−6/+6), RFC1 80G > A, RFC1−43 T > C, FPGS 1994G > A, GGH 452C > T, GGH−401C > T, ABCB1 3435C > T, ABCC2 IVS 23 + 56 T > C, and ABCG2 914C > A polymorphisms in 159 Japanese RA patients are summarized in Table 3. The proportions of genotypes at each site for their transporter carrier proteins and enzymes were generally in agreement with Hardy-Weinberg equilibrium, following Mendelian principles. Only three genotype combination patterns occurred for RFC1 80G > A and RFC1−43 T > C, consisting of the 80G/G,−43 T/T genotype, 80G/A,−43 T/C genotype, and 80A/A,−43C/C genotype, respectively and a strong linkage disequilibrium was observed in Japanese RA patients. Allelic frequencies of TYMS 3'-UTR−6 > +6 were 38% in men and 32% in women for the +6 allele, and a significant gender difference in TYMS 3'-UTR−6 > +6 (*p* = 0.0064, chi-square test) was seen, but the proportions of genotypes in men were not in agreement with Hardy-Weinberg equilibrium (*p* = 0.003, chi-square test). Gender differences in the distribution of other genotypes were not observed (Table 3), although a tendency toward gender differences was seen in the distribution of MTHFR 1298 A > C (*p* = 0.0736, chi-square test).

### Comparison of allelic frequencies between healthy Japanese adults and RA patients

The distributions of MTHFR 1298A > C, TYMS 3'-UTR (−6/+6), RFC1 80G > A, RFC1−43 T > C, FPGS 1994G > A, GGH 452C > T, GGH−401C > T, ABCB1 3435C > T, ABCC2 IVS 23 + 56 T > C, and ABCG2 914C > A polymorphisms in 299 healthy Japanese healthy adults and 159 Japanese RA patients are summarized in Table 3. There were no statistically significant differences in allelic frequencies between the healthy and RA groups.

## Discussion

We comprehensively investigated the allelic frequencies in the gene polymorphisms of enzymes and transporter proteins including MTHFR, TYMS, RFC1, FPGS, GGH, ABCB1, ABCC2 and ABCG2, which affect MTX pharmacokinetics and the therapeutic response to it, between a healthy Japanese population and Japanese RA patients to obtain the fundamental data for the personalized MTX therapy for RA.

Characteristics of MTX-related enzyme gene and transporter polymorphisms are summarized in Table 4. In the comparison of allelic frequencies in ethnic and/or population groups in healthy adults and RA patients, the frequency of the MTHFR1298 C allele in our Japanese study was significantly lower than in Caucasian [4, 7, 8], but higher than in Africans [4]. The MTHFR gene is associated with the generation of 5-methyl THF, the MTHFR 1298A > C polymorphism decreases MTHFR activity, and is thereby associated with MTX efficacy [41, 42] and toxicity [26, 43]. In terms of the effect of each SNP on MTX efficacy and toxicity, it is considered to be the greatest in Caucasians [4, 7, 8], followed by Japanese and Africans [4]. However, currently there are few data comparing Japanese with Africans in relation to the efficacy and toxicity of MTX treatment. As such one example, Hughes et al. [26] reported that there was an association between scores of MTX toxicity and the rs4846051 C allele, that is, a higher mean toxicity score among African-Americans than among Caucasians, and haplotypes containing this allele in African-Americans, but not in Caucasians.

**Table 4:** Summarized characteristics of MTX-related enzyme gene and transporter polymorphisms

Gene	SNP ID	SNP allele	Effects on gene product/ enzyme	Clinical significance
MTHFR	rs1801131	1298 A > C	Decreases MTHFR activity	Associated with MTX efficacy, not associated with MTX toxicity, or associated with MTX toxicity
TYMS	rs16430	3'-UTR - 6/6	Decreases TYMS mRNA expression	Associated with MTX toxicity
RFC1	rs1051266	80 G > A	Affects transcriptional activity of RFC1 gene and MTX entry into cells	Increases MTX-PG concentrations and good clinical response to MTX treatment
	rs1131596	−43 T > C	Decreases the expression of RFC1 protein	Unknown
FPGS	rs10106	1994 G > A	Associated with FPGS mRNA expression	Not associated with MTX efficacy/toxicity, or poor response to MTX treatment
GGH	rs11545078	452 C > T	Associated with lower GGH activity, greater accumulation of long-chain MTX-PGs	Not associated with MTX efficacy/toxicity
	rs3758149	−401 C > T	Associated with greater GGH promotor activity, decrease polyglutamation	Affects MTX toxicity
ABCB1	rs1045642	3435 C > T	Decreases stability/expression of mRNA, reduces the activity of efflux transporters	Associated with MTX efficacy
ABCC2	rs4148396	IVS23 + 56 T > C	Affects ABCC2 enzyme activity and MTX efflux from cell	Associated with MTX toxicity (adverse GI effects)
ABCG2	rs2231142	914 C > A	Increases MTX-PG1/MTX-PG2 concentrations	Associated with MTX toxicity

*MTX*: methotrexate, *MTHFR*: methylenetetrahydrofolate reductase, *TYMS*: thymidylate synthase, *RFC1*: reduced folate carrier 1, *FPGS*: folypolyglutamayl synthase, *GGH*: γ-glutamyl hydrolase, *ABCB1*: ATP-binding cassette sub-family B member 1, *ABCC2*: ATP-binding cassette subfamily C member 2, *ABCG2*: ATP-binding cassette sub-family G member 2, *GI*: gastrointestinal

The frequency of RFC1 80 A and RFC1−43C alleles in the present Japanese study was found to be higher than in Caucasian healthy adults [4, 7, 8, 11] and RA patients [24, 34–36]. The present study found it to be lower in Japanese than in African healthy adults [4]. RA patients with the RFC1 80 A/A genotype had increased MTX-PG concentrations in RBCs [9, 41], and the mean MTX-PG concentration in RBCs in RA patients with that genotype was reported to be 3.4-fold greater than in other genotypes [44]. Those patients showed a good clinical response to MTX treatment [35]. On the other hand, the−43 T > C change decreased the expression of RFC1 protein in patients with RA [34]. The directions in MTX-PG influx between RFC1 80 G > A and−43 T > C are completely opposite. To clarify the contributions of these two genotypes to MTX-PG concentrations in RBCs, it will be necessary to measure the concentrations in RFC1 80G > A and RFC1−43 T > C RA patients.

The frequency of the FPGS1994 A allele in our Japanese study was significantly lower than reported in Caucasian [4] and Africans [4]. FPGS 1994 A > G polymorphism reported to have no effect on MTX efficacy or toxicity [45], although there was an association between FPGS mRNA expression in peripheral blood mononuclear cells and a poor response to MTX in RA patients with that genotype [46]. Therefore, it is unknown whether the FPGS 1994 A > G polymorphism is associated with the inter-individual difference in MTX efficacy or toxicity.

For the frequency of the GGH 452 T allele, our Japanese study found it to be significantly lower than reported in Caucasian healthy individuals [4] and was the almost same as in Caucasian RA patients [27, 37, 38]. The GGH−401 T allelic frequency in our study was also lower than in HapMap-JPT [4] and in Caucasians [4], but it was higher than in Africans [4]. The GGH gene is involved in reversing polyglutamation by removing glutamate moieties, and a specific functional SNP (452C > T) in the human GGH gene is associated with lower catalytic activity and greater accumulation of long-chain MTX-PGs in leukemia cells of patients treated with high-dose MTX [47]. The−401C > T mutation resulting in greater GGH promotor activity increases the hydrolytic activity of MTX-PGs and decreases polyglutamation in the−401TT genotype compared with the−401CC or CT genotype [44]. Thus, MTX might be more effective in Caucasian than in Japanese RA patients at the same dose, when we speculate on the intracellular MTX-PG concentration.

The frequency of the ABCB1 3435 T allele in our study was lower than reported in Caucasian [4] but higher than in African healthy adults [4]. Conversely, the ABCC2 IVS23 + 56 C allelic frequency was higher in our study than reported in Caucasians [4] but lower than in Africans [4]. We also found a higher frequency of the ABCG2 914 A allele in our study than reported in Caucasians [4] and Africans [4]. The ABCB1 3435C > T mutation resulting in decreased stability and expression of mRNA reduces the activity of efflux transporters, may affect P-glycoprotein function and MTX efflux from cells [48], and is associated with MTX efficacy. It was reported that the length of time before it became necessary to reduce the MTX dose or discontinue administration due to the occurrence of toxicity was 2 months in patients with the T/T genotype, 23 months in those with the T/C genotype, and 29 months in those with the C/C genotype, and the time period was significantly correlated with genotype [40]. Therefore, it is considered that the ABCC2 IVS23 + 56 T > C mutation may affect ABCC2 enzyme activity involved in MTX efflux from cells [40]. The ABCG2 914C > A mutation was reported to increase MTX-PG_1_ and MTX-PG_2_ concentrations in RA patients [27]. It is considered that MTX efficacy is the highest in Caucasians, followed by Japanese and Africans, from the viewpoint of ABCB1 3435C > T; conversely, it is the highest in Caucasians, followed by Japanese and Africans, from the viewpoint of ABCC2 IVS 23 + 56 T > C; and the highest in Japanese, followed by Caucasians and then Africans, from the viewpoint of ABCG2 914 C > A. However, we need to investigate the net contribution in MTX-PG efflux among three ABC transporters polymorphism.

There were no previous reports of RFC1−43 T > C, FPGS 1994 G > A, GGH−401 C > T, and ABCC2 IVS23 + 56 T > C in a healthy Japanese population except for HapMap-JPT and RFC1−43 T > C, FPGS 1994 G > A, and ABCC2 IVS23 + 56 T > C in Japanese patients with RA for the evaluation of allelic frequency. Therefore, this is the first comprehensive report on these genetic polymorphisms in healthy Japanese adults and Japanese RA patients.

When comparing healthy Japanese adults with Japanese RA patients, there were no significant differences in allelic frequencies of the MTHFR 1298A > C, TYMS 3'-UTR (−6/+6), RFC1 80G > A, RFC1−43 T > C, FPGS 1994G > A, GGH 452C > T, GGH−401C > T, ABCB1 3435C > T, ABCC2 IVS 23 + 56 T > C, and ABCG2 914C > A polymorphisms in 299 healthy Japanese adults and 159 Japanese RA patients. Therefore, we confirmed that the allelic frequencies of those SNPs in healthy Japanese adults were almost the same as in Japanese patients with RA in this study.

The allelic frequencies of SNPs in Japanese RA patients in this study were in agreement with Hardy-Weinberg equilibrium and were considered representative of the Japanese population as a whole. Chatzikyriakidou et al. [34] reported a strong linkage disequilibrium between RFC1 80 G > A and RFC1−43 T > C genotypes for only three patterns in the combination of the RFC1 80 G > A with RFC1−43 T > C genotype, that is, 80G/G and−43 T/T, 80G/A and−43 T/C, and 80A/A and−43C/C in Greek patients with RA. In agreement with the results of Chatzikyriakidou et al. [34], we also confirmed that the three patterns in the combination of the RFC1 80 G > A with RFC1−43 T > C genotype had a strong linkage disequilibrium between RFC1 80 G > A and RFC1−43 T > C genotypes in healthy Japanese adults and RA patients.

Gender differences in allelic frequencies of RFC1 80G > A, RFC1−43 T > C, and ABCC2 IVS23 + 56 T > C in healthy Japanese adults and TYMS 3'-UTR−6 > +6 in Japanese RA patients were observed. Meanwhile, Kameda et al. [48] reported that the tender joint count, swollen joint count and decrease in serum C-reactive protein (CRP) levels as indices of MTX treatment response in Japanese patients with RA were better in men than in women and gender differences in the MTX therapeutic response were suggested in Japanese RA patients. Currently, however, we cannot clearly explain the reason for the gender differences in these allelic frequencies between healthy Japanese adults and Japanese RA patients, but Hardy-Weinberg disequilibrium in the distribution of those SNPs in either men or women may be one factor responsible for the differences.

This study had several limitations: 1) There were only a small number of reports in the literature allowing comparisons of ethnic and/or population groups, because most did not clearly describe the ethnicity/race and/or population groups studied. These were assumed based on the country of residence of the lead author, and therefore might have misclassified ethnicity/race and/or population groups. 2) We collected allelic frequency data from the HapMap Project for comparisons with our Japanese study results in cases when sufficient data could not be retrieved from the literature search. Although the HapMap data contained the same rs numbers, they included different ss numbers. 3) Several papers used for comparison with our results did not report the rs numbers for ethnic/race and/or population groups. Therefore, our comparisons may not be completely accurate in using the same rs numbers of SNPs. 4) All SNPs studied here could not be compared among ethnic/race and/or population groups due to a lack of data. This study may also include mixed ethnicities/races in a population group that we treated as a single national population, because that was assumed based on the lead author’s country of residence. 5) The distributions of several SNPs in the ethnic/population group in healthy adults and RA patients were also not in agreement with Hardy-Weinberg equilibrium. 6) We did not evaluate allelic frequencies using combinations of SNPs such as diplotypes or haplotypes in our Japanese population. 7) There are few studies of MTX pharmacokinetics among ethnic/population groups. We also did not investigate MTX-PGs pharmacokinetics among ethnic/population groups by comparing the genotypes of MTX-related SNPs to clarify interethnic variations in MTX pharmacokinetics and therapeutic response. This is the greatest limitation in this study.

## Conclusion

This study identified allelic frequencies in ethnic and/or population groups compared with healthy Japanese and RA patients. The differences in frequencies may be variables in the interethnic variations in MTX response. Further study is needed to confirm the association of these genetic/phenotypic factors and clinical outcomes and whether these SNPs may be useful in determining personalized MTX therapy for RA.

## Abbreviations

3'-UTR: 3'-untranslated region
ABCB1: ATP binding cassette subfamily B member 1
ABCC2: ATP-binding cassette sub-family C member 2
ABCG2: ATP-binding cassette sub-family G member 2
FPGS: Folypolyglutamyl synthase
GGH: γ-glutamyl hydrolase
IVS: Intervening sequence
MTHFR: Methylenetetrahydrofolate reductase
RFC1: Reduced folate carrier 1
TYMS: Thymidylate synthase
